# Comparative Mechanisms of Different *Bifidobacteria* in Combating Pathogen Infection and Prolonging the Lifespan in *Caenorhabditis elegans*

**DOI:** 10.3390/microorganisms13122861

**Published:** 2025-12-16

**Authors:** Xueer Wang, Shixiang Wu, Wen Zhao, Zhaozhong Zeng, Jian He, Hanglian Lan, Bing Fang, Haina Gao, Yue Liu, Jingyu Li, Weilian Hung, Ming Zhang

**Affiliations:** 1 School of Food and Health, Beijing Technology and Business University, Beijing 100048, China; 13750941877@163.com (X.W.); 18811068596@163.com (S.W.); gaohaina103@126.com (H.G.); liuyue2132@163.com (Y.L.); ljy110808@163.com (J.L.); 2 Key Laboratory of Precision Nutrition and Food Quality, Institute of Nutrition and Health, China Agricultural University, Beijing 100193, China; zhaowen@yili.com (W.Z.); bingfang@cau.edu.cn (B.F.); 3 National Technology Innovation Center for Dairy, Hohhot 010100, China; zengyuanyi2021@163.com (Z.Z.); hejian@yili.com (J.H.); lanhanglian@yili.com (H.L.); 13774299154@163.com (W.H.)

**Keywords:** *Bifidobacteria*, lifespan-extending, pathogen resistance, *Caenorhabditis elegans*

## Abstract

The research employed *Caenorhabditis elegans* to compare the anti-infection and lifespan-extending properties of *Bifidobacterium*. The results demonstrated that BL-99 and YLGB-1496 intervention improved the nematodes’ resistance to *Staphylococcus aureus* infection, resulting in lifespan extensions of 5.90% and 14.38%, respectively, accompanied by the alleviation in the decline of pharyngeal pumping rate and locomotor capacity. Furthermore, both probiotic strains significantly extended the mean lifespan of nematodes by 10.96% and 12.14%, and significantly alleviated pharyngeal pumping and locomotion. Importantly, BL-99 and YLGB-1496 have different underlying mechanisms of action. Transcriptomic analyses indicated that the BL-99 strain enhanced nematode resistance to Gram-positive pathogens through the upregulation of lysozyme, saposin-like antimicrobial peptides, and c-type lectin family genes. Conversely, YLGB-1496 improved the epidermal permeability barrier by upregulating genes involved in collagen synthesis and assembly. Overall, this study provides novel insights into the species-specific effects of *Bifidobacteria* on pathogen resistance and lifespan extension.

## 1. Introduction

*Bifidobacterium*, a genus of bacteria prevalent in the human gut, has garnered increasing attention for its potential contributions to human health and longevity. Recent studies have indicated that a positive correlation between *Bifidobacterium* presence and lifespan [1,2,3]. Furthermore, research has demonstrated that *Bifidobacterium* levels decline with age, potentially contributing to the onset of age-related diseases [4]. Additionally, the bifidogenic effect of 2-fucosyllactose, a prebiotic, has been shown to be age-specific, promoting the growth of different *Bifidobacterium* species depending on the age group [5]. These findings underscore the importance of considering age-related changes in the gut microbiome when developing targeted interventions.

*Bifidobacterium* has been recognized as a dominant constituent of the gut microbiota in both infants and centenarians, indicating its potential role in modulating immune responses and attenuating inflammation-related cytokines, which are essential for healthy aging [6]. This observation was consistent with research findings that *Bifidobacterium* species can enhance immune function and mitigate oxidative stress, both critical factors in delaying the aging process [7]. Additionally, *Bifidobacterium* can competitively inhibit the colonization of pathogenic bacteria, thereby reducing the proliferation of harmful microorganisms in the intestine [8]. *Bifidobacterium* can upregulate the expression of tight junction proteins, thereby enhancing the barrier integrity of intestinal epithelial cells [9]. It can also alleviate inflammation caused by infection by promoting the secretion of anti-inflammatory cytokines and inhibiting the expression of pro-inflammatory factors [10].

Research into the genomic diversity of *Bifidobacterium* species has unveiled substantial differences in their functional capabilities, which may play a crucial role in their health-promoting effects [11]. Ménard (2012) conducted a comparative analysis of the immunomodulatory functions of *Bifidobacterium* across four species and discovered that their capacity to stimulate the immune system is both species- and strain-specific [12]. Notably, species-specific variations are prevalent in the widely utilized strains of *Bifidobacterium animalis* and *Bifidobacterium longum*, particularly in the context of alleviating gastrointestinal, immune, and infectious diseases [13]. *Bifidobacterium longum* subsp. *infantis* was distinguished by its extensive repertoire of genes associated with the utilization of human milk oligosaccharides, potentially conferring a competitive advantage within the gut microbiota and enhancing its probiotic efficacy [14]. Furthermore, the presence of bacteriocin gene clusters in *Bifidobacterium longum* subsp. *infantis* suggested a role in maintaining gut microbiota equilibrium and preventing pathogenic colonization, thereby supporting its potential in promoting longevity [15]. *Bifidobacterium animalis* was recognized for its high tolerance to acidic, bile, and oxygen-rich environments [16].This species-specific adaptation highlights the importance of understanding the ecological and metabolic strategies of different *Bifidobacterium subspecies*, as they may offer insights into their roles in promoting longevity [17].

Therefore, the differences in the efficacy and mechanism of action of bifidobacteria in prolonging lifespan and anti-infection have become an emerging research field. For this reason, we chose *Caenorhabditis elegans* as the model organism.

As a model organism, *Caenorhabditis elegans* (*C. elegans*) is well-suited for studies on anti-aging due to its advantages, including ease of cultivation, short life cycle, high reproducibility, and a fully sequenced genome [18]. Certain aging pathways and mechanisms have been shown to be conserved between *C. elegans* and humans. The genetic pathways involved in nematode aging, such as insulin signaling and the AMPK pathway, are highly conserved when compared to humans [19,20]. Research has demonstrated that mutations in the *daf-2* gene can significantly extend the lifespan of nematodes by reducing insulin signal transduction through the *daf-16*/FOXO transcription factors, which are also considered to contribute to human longevity [21]. Consequently, *C. elegans* can serve as a model to investigate the anti-aging capabilities and mechanisms of probiotics.

*Bifidobacterium animalis* subsp. *lactis* BL-99 (BL-99) is a probiotic isolated from the gut of healthy infants [22], while *Bifidobacterium longum* subsp. *infantis* YLGB-1496 (YLGB-1496) was derived from human breast milk [23]. The objective of this study was to compare the lifespan-extending effects of *Bifidobacterium* strains in *C. elegans* and to elucidate the mechanistic differences underlying lifespan prolongation by distinct *Bifidobacterium* species through transcriptomic analysis.

## 2. Materials and Methods

### 2.1. Bacterial Strains and Nematodes Preparation

BL-99 and YLGB-1496 strains were procured from the National Technology Innovation Center for Dairy in Hohhot, China (Table S1). The bacterial cells were cultured in MRS broth at 37 °C under anaerobic conditions. Following overnight incubation, the cells were harvested, washed with phosphate-buffered saline (PBS) for three times, and resuspended in sterile PBS (pH 7.4) to achieve a concentration of 1 × 10^8^ CFU/mL.

*Staphylococcus aureus* ATCC25923 (*S. aureus*) served as the Gram-positive pathogen and was cultured aerobically in LB broth at 37 °C for 24 h. The bacterial cells were then suspended in 0.25 mL of PBS buffer to a concentration of 1 × 10^8^ CFU/mL, and 60 μL of this suspension was spread onto nematode growth medium (NGM) in 6.0 cm-diameter plates for nematode feeding.

*Escherichia coli* OP50 (OP50) and the *Caenorhabditis elegans* (*C. elegans*) Bristol N2 strain were supplied by Professor Yanling Hao’s laboratory (Table S1). OP50 was cultured anaerobically in LB broth at 37 °C for 12 h and used as the standard feed for nematode cultivation. Nematodes were maintained and propagated on NGM following established protocols. Nematodes are synchronized before the experiment begins. After synchronization, L4 stage nematodes were transferred to modified NGM medium containing 50 mg/mL 5-fluoro-2′-deoxyuridine (FUdR), which is utilized to inhibit nematode oviposition. After cell harvest, OP50 suspended in 0.25 mL PBS buffer (pH 7.4, sterile) to 1 × 10^8^ CFU/mL, and 60 μL of suspension was then spread on NGM to feed the worms. In order to prevent pollution and maintain bacterial activity, OP50, *S. aureus* and probiotics are added to the plate in the form of concentrated solution, and the feeding solution and plate are renewed every 2–3 days. Nematodes eat fresh bacterial mixture every day.

### 2.2. Experimental Design

**Figure 1:**
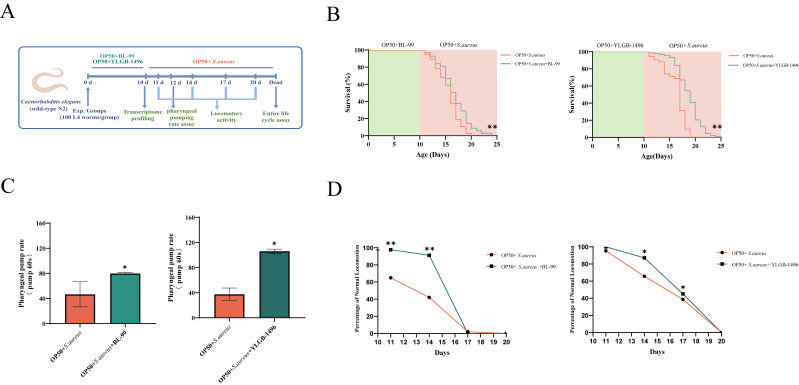
**BL-99 and YLGB-1496 enhanced the tolerance of *****C. elegans***** to *****S. aureus***** infection.** (**A**) Experimental flowcharts. (**B**) Survival curves of *C. elegans* that were fed OP50+BL-99, OP+YLGB-1496 or only OP50 for 10 days before infection with *S. aureus.* OP50*+S. aureus* worms, *n* = 93; OP50+BL-99*+S. aureus* worms, ** *p* < 0.01, one-way ANOVA, *n* = 71; OP50*+S. aureus* worms, ** *p* < 0.01, one-way ANOVA, *n* = 92, OP50+YLGB-1496*+S. aureus* worms, *p* < 0.01, one-way ANOVA, *n* = 88. (**C**) The pharyngeal pumping rate from 12th N2 worms grown on OP50*+*BL-99 or OP50+YLGB-1496 for 10 days before infection with *S. aureus* compared to OP50*+S. aureus* worms (* *p* < 0.05, one-way ANOVA, *n* = 20). (**D**) The Class A of locomotory activity of different groups *C. elegans* on the 11th, 14th, 17th and 20th day (* *p* < 0.05, ** *p* < 0.01, Chi-squared test, *n* ≥ 90 worms).

This experiment is mainly divided into two parts. The first part is about the protective effect of *Bifidobacteria* against *S. aureus* infection in nematode (Figure 1A). The young adult worms (0 days old) were assigned to either a control group that was fed OP50 or to a group that was fed BL-99 or YLGB-1496 for 10 days. The nematodes in the control group were only fed with OP50 for the first 10 days, and on the 11th day, OP50 and *S. aureus* were co-cultured without adding any probiotics. The concentration of *Bifidobacterium* BL-99 and YLGB-1496 bacterial solution is 1 × 10^8^ CFU/mL, which is mixed with OP50 in a 1:1 ratio and evenly spread on an agar plate. The L4 synchronized worms were then transferred onto *S. aureus* lawns. The concentration of *S. aureus* (1 × 10^8^ CFU/mL) was mixed with OP50 in a 1:1 ratio24. Each group was incubated at 20 °C, and their numbers were recorded daily until all worms died. The second component examines the impact of *Bifidobacteria* on the lifespan of nematodes. The nematodes in the control group were only fed with OP50 and no probiotics were added. Young adult worms, aged 0 days, were assigned to a control group fed with OP50, or to groups fed with BL-99 or YLGB-1496. L4 worms were then transferred onto OP50 lawns. The concentrations of BL-99 and YLGB-1496 were mixed with OP50 in a 1:1 ratio. Each group was incubated at 20 °C, with daily recordings of worm numbers until all worms had died. Sterility was kept during the co-culture of nematodes and bacteria.

### 2.3. Lifespan Measurement

For lifespan measurement, refer to the previously established method [24], where nematode eggs were retrieved from adult *C. elegans* following exposure to sodium hypochlorite/sodium hydroxide solution. The synchronized L4-stage nematodes were transferred to 35 mm NGM plates containing 50 mg/mL FUdR, and their numbers were recorded daily. In the BL-99 or YLGB-1496 treatment groups, *C. elegans* N2 were administered a diet of 1 × 10^8^ CFU/mL BL-99 or YLGB-1496 for a duration of 10 days. Subsequently, each group was maintained at 20 °C until all nematodes had expired. In evaluating the condition of *C. elegans*, any nematode exhibiting no movement was classified as deceased. All experiments were conducted in triplicate.

### 2.4. Pharyngeal Pumping Rate

The pharyngeal pumping rate assay was performed as described by Zhao et al. [25]. To evaluate the frequency in both the control and treatment groups (BL-99 and YLGB-1496), a total of 10 nematodes per biological replicate were placed onto their respective NGM plates seeded with OP50. The pharyngeal pumping rate, defined as the number of contractions per 10 s, was observed at specific time intervals using a stereo microscope.

### 2.5. Motility Assessment

The motility of *C. elegans* was assessed on days 11, 14, 17, and 20. The motility classification was determined according to previously established methods [26], with Class A assigned to nematodes that moved spontaneously and smoothly, leaving sinusoidal and symmetric tracks, and Class C designated for nematodes that only moved their head or tail when prodded with a soft wire. Class B was assigned when the behavioral class was intermediate between A and C. Class D was designated when nematodes dead.

### 2.6. Transcriptomic Analysis

Following 10 days treatment, the nematodes were cleaned three times using PBS, and then total RNA was extracted by QIAzol Lysis Reagent (Qiagen, Hilden, Germany, 79306). Each group has three replicates, and each replicate contains more than 1000 nematodes. Library establishment and RNA sequencing were performed by Majorbio (Shanghai, China). The library preparations were established by Illumina^®^ Stranded mRNA Prep Ligation and were sequenced on NovaSeq Xplus (Illumina, San Diego, CA, USA). Quality control of the sequencing data was conducted using fastp (*Shifu Chen. fastp 1.0*). Then, the differentially expressed genes (DEGs) between groups, and GO enrichment analyses of DEGs were conducted on a Majorbio Cloud Platform (https://www.majorbio.com/，accessed on 5 September 2025). DEGs were determined by *p* < 0.05, log_2_(fold change) > 1.5.

### 2.7. Statistical Analysis

The data are presented as mean ± standard error. Data analysis was performed using GraphPad 10.0 software. The percentage increase in lifespan is expressed as a percentage increase. All other data were analyzed for significance using one-way ANOVA and Tukey’s multiple comparisons. A *p*-value < 0.05 indicates a significant difference, and a *p*-value < 0.01 indicates an extremely significant difference.

## 3. Results

### 3.1. BL-99 and YLGB-1496 Enhanced the Tolerance of C. elegans to S. aureus Infection

In this study, we initially examined the protective effects of BL-99 and YLGB-1496 against *S. aureus* infection in nematodes. As illustrated in Figure 1B, nematodes fed with BL-99 and YLGB-1496 exhibited improved survival rates compared to those fed with OP50 following *S. aureus* infection. Specifically, BL-99 increased the mean lifespan of nematodes from 15.09 days to 15.98 days, representing a 5.9% enhancement, while YLGB-1496 extended the mean lifespan from 16.78 days to 19.19 days, indicating a 14.4% improvement .

The pharyngeal pumping rate, a key indicator of nematode health during feeding, was significantly impacted by *S. aureus* infection, reducing to approximately 40 times per minute by day 12 (Figure 1C). In the BL-99 intervention group, the pharyngeal pumping frequency increased by 71.33% compared to the *S. aureus* group , whereas the YLGB-1496 intervention group exhibited an increase of 183.93%. Therefore, the YLGB-1496 intervention group showed better improvement in pharyngeal pumping ability than the BL-99 group.

Locomotory activity, another critical measure of nematode health, was evaluated on days 11, 14, 17, and 20. As illustrated in Figure 1D, infection with *S. aureus* resulted in a significant reduction in the locomotory activity of nematodes. In contrast, nematodes in the BL-99-fed group demonstrated markedly higher levels of coordinated sinusoidal locomotion (classified as class A) compared to the control group on days 11 and 14 . Concurrently, the intervention with YLGB-1496 improved nematode motility, with the effects persisting until day 17 . These findings suggested that both BL-99 and YLGB-1496 enhanced the tolerance of *C. elegans* to *S. aureus* infection, with YLGB-1496 exhibiting better efficacy. From the proportion of A-class motor ability on the 17th day, the YLGB-1496 group showed better improvement in nematode ability.

### 3.2. BL-99 and YLGB-1496 Prolonged the Lifespan of C. elegans

**Figure 2:**
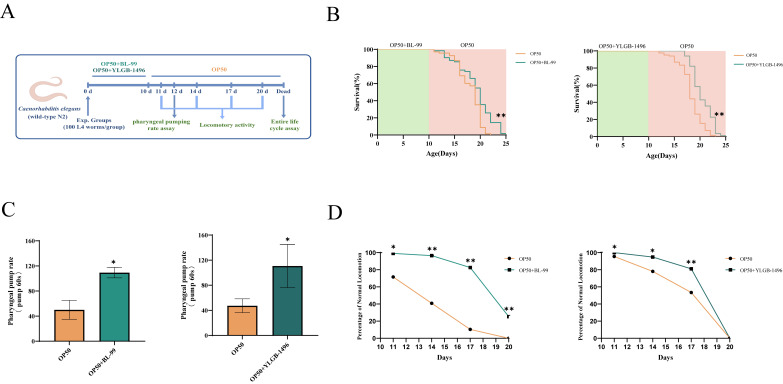
**BL-99 and YLGB-1496 prolonged the lifespan of *****C. elegans.*** (**A**) Experimental flowcharts. (**B**) Survival curves of *C. elegans* that were fed OP50+BL-99, OP+YLGB-1496 or only OP50 for 10 days. OP50 worms, *n* = 83, OP50+BL-99 worms, *n* = 71 and OP50 worms, *n* = 85, OP50+YLGB-1496 worms, *n* = 84, ** *p* < 0.01, one-way ANOVA. (**C**) The pharyngeal pumping rate from 12th N2 worms grown on OP50+BL-99 or OP50+YLGB-1496 for 10 days compared to OP50 worms (* *p* < 0.05, one-way ANOVA, *n* = 20). (**D**) The Class A of locomotor activity from wild-type N2 worms grown on OP50 andOP50 BL-99, YLGB-1496 was measured on the 11th, 14th, 17th and 20th day (* *p* < 0.05, ** *p* < 0.01, Chi-squared test, *n* ≥ 90).

In the aforementioned study, we identified that the two *bifidobacteria* strains not only combated pathogenic bacterial infections but also potentially extended the lifespan of nematodes. To substantiate this observation, we conducted experiments using uninfected nematodes to assess the lifespan-prolonging effects of BL-99 and YLGB-1496 (Figure 2B). Compared to nematodes fed solely on OP50, those treated with BL-99 and YLGB-1496 exhibited a significant increase in lifespan (*p* < 0.05). Specifically, BL-99 extended the mean lifespan of nematodes from 16.73 days to 18.73 days, representing an increase of 10.96%, while YLGB-1496 extended the mean lifespan from 18.64 days to 20.90 days, corresponding to a 12.14% increase.

We evaluated two common indicators of aging: pharyngeal pumping rates and locomotor abilities, under the interventions of BL-99 and YLGB-1496, to assess the overall health status of nematodes. As the nematodes aged naturally, a decline in pharyngeal pumping rates was observed in the OP50-fed group after 10 days. In contrast, nematodes fed with BL-99 and YLGB-1496 exhibited significantly higher pharyngeal pumping rates compared to the control group, with increases of 118.67% and 133.80%, respectively (*p* < 0.05, Figure 2C).

Locomotor activity was also assessed on days 11, 14, 17, and 20. As depicted in Figure 2D, the locomotor abilities of the nematodes diminished with natural aging. The BL-99-fed group demonstrated superior locomotor activity compared to the control group throughout the testing period . Additionally, the YLGB-1496 intervention improved nematode motility, with effects lasting until day 17 . These findings suggested that BL-99 and YLGB-1496 significantly delayed the natural aging process in nematodes and enhanced their feeding and motility capabilities.

### 3.3. Transcriptional Analysis of C. elegans After Feeding with BL-99 and YLGB-1496

**Figure 3:**
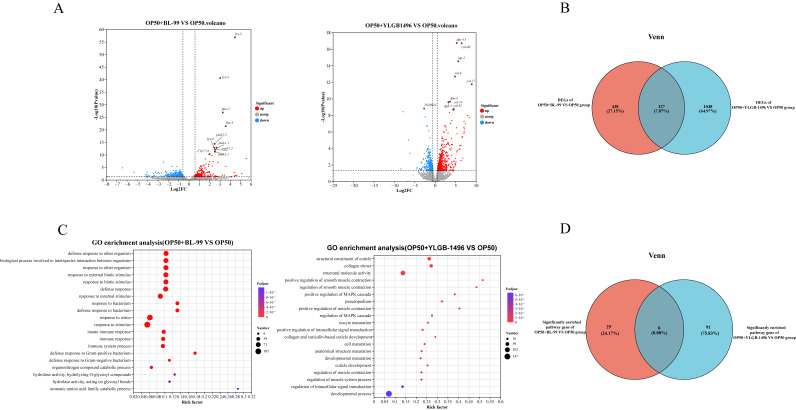
**Transcriptional analysis of *****C. elegans***** after feeding with BL-99 and YLGB-1496.** (**A**) Gene expression profiles of OP50+BL-99 or OP50+YLGB-1496 *C. elegans* expression werecompared with OP50 worms. Volcano plot showing the up- and down-regulated genes in OP50+BL-99 or OP50+YLGB-1496 groups with OP50 *C. elegans* (*p* < 0.01 and Fold Change > 1.5). (**B**) The Venn plot of differential genes between OP50+BL-99 and OP50+YLGB-1496 *C. elegans*. (**C**) GO-BP enrichment of the up-regulated genes in OP50+BL-99 and OP50+YLGB-1496. Some GO terms were specified. (**D**) The Venn plot of the GO gene set: OP50+BL-99 groups enrichment pathway for defense response to Gram-positive bacterium. OP50+YLGB-1496 groups enrichment pathway for structural molecular activity. Each group has three replicates, *n* > 1000.

The aforementioned results demonstrated that nematodes subjected to a 10-day regimen of BL-99 and YLGB-1496 exhibited notable extensions in lifespan and increased resistance to pathogenic bacterial infections. To investigate the impact of these bifidobacterial supplements on nematodes, we utilized transcriptomic analysis to conduct a comprehensive assessment of gene expression following the 10-day feeding period with BL-99 and YLGB-1496. Differential gene expression (DGE) analysis was conducted to compare the *Bifidobacteria* groups with the OP50 group (*p* < 0.05, log_2_(fold change) > 1.5). As illustrated in Figure 3A, the BL-99 and YLGB-1496 groups exhibited 522 and 1138 differentially expressed genes, respectively, when compared to the OP50 group. Specifically, the BL-99 group showed 235 significantly upregulated and 287 significantly downregulated genes, while the YLGB-1496 group displayed 737 significantly upregulated and 401 significantly downregulated genes.

To elucidate the similarities and differences in the effects of BL-99 and YLGB-1496 on nematodes, a Venn diagram analysis was conducted to identify overlapping DEGs. As depicted in Figure 3B, only 127 DEGs were common to both treatment groups, representing 7.87% of the total. The BL-99 intervention group uniquely expressed 438 DEGs, whereas the YLGB-1496 intervention group uniquely expressed 1048 DEGs. This finding suggested that the beneficial mechanisms of BL-99 and YLGB-1496 against nematodes may differ.

To explore the mechanistic differences underlying the lifespan-prolonging effects of BL-99 and YLGB-1496, Gene Ontology (GO) enrichment analysis was conducted. The GO functional enrichment analysis revealed that, in comparison to the OP50 group, the BL-99 treatment group was predominantly enriched in pathways related to response to bacterium, response to stimulus, response to stress, and immune response, all of which are closely associated with the innate defense system (Figure 3C). Notably, the defense response to Gram-positive bacterium pathway exhibited a high rich factor and included 29 DEGs.

In contrast, the YLGB-1496 intervention group showed significant enrichment in pathways such as collagen trimer, structural constituent of cuticle, and structural molecule activity (Figure 3C). Specifically, the structural molecule activity pathway included 91 DEGs. It is inferred that the YLGB-1496 intervention may enhance the integrity of the nematode cuticle, thereby providing increased protection against pathogenic bacteria.

The Venn diagram of the GO enrichment analysis was conducted to identify overlapping pathways between BL-99 and YLGB-1496 treatments. Notably, there were no shared pathways among the 29 pathways significantly enriched in the BL-99 group and the 91 pathways significantly enriched in the YLGB-1496 group. This observation further substantiated the hypothesis that the mechanisms through which these two probiotic strains exert beneficial effects on nematodes are likely distinct.

### 3.4. BL-99 Intervention Enhanced the Defense Response to Gram-Positive Bacteria of Nematode

**Figure 4:**
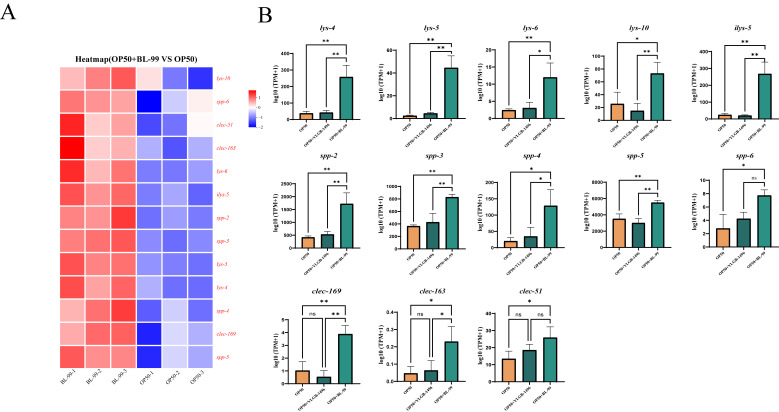
**BL-99 intervention enhanced the defense response to Gram-positive bacteria of nematode.** (**A**) Up regulation gene heat map in GO terminology of defense response of Gram-positive bacteria in BL-99 feeding group. (**B**) Bl-99 feeding group significantly increased gene expression in OP50, BL-99, YLGB-1496 feeding groups. Each group has three replicates, *n* > 1000, * *p* < 0.05, ** *p* < 0.01.

The pathway related to the defense response to Gram-positive bacteria was enriched, highlighting genes activated in response to the presence of a Gram-positive bacterium, which function to protect the organism. Subsequently, we compared the major DEGs within this pathway between the BL-99-treated group and the control group. As illustrated in Figure 4A, relative to the OP50-treated group, 13 significantly upregulated genes (indicated in red) were identified in the BL-99-treated group (*p* < 0.05). These included five genes associated with lysozyme activity (*lys-4*, *lys-5*, *lys-6*, *lys-10*, and *ilys-5*), three genes associated with C-type lectin (*clec-51*, *clec-163*, and *clec169*), and five genes associated with saposin-like antimicrobial proteins (*spp-2*, *spp-3*, *spp-4*, *spp-5*, and *spp-6*). All these genes are implicated in mediating host defense and play a crucial role in the organism’s protective mechanisms.

### 3.5. YLGB-1496 Intervention Enhanced the Structural Molecule Activity of Nematode

**Figure 5:**
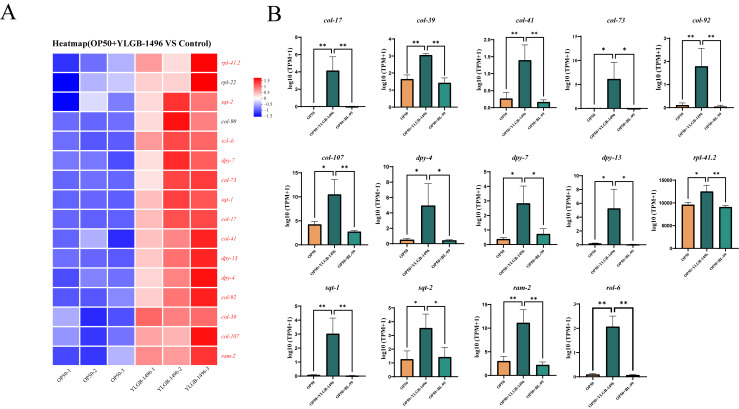
**YLGB-1496 intervention enhanced the structural molecule activity of nematode**. (**A**) Up regulation gene heat map in GO terminology of structural molecule activity in YLGB-1496 feeding group. The colors in the figure represent the standardized expression levels of the gene in each sample, with red indicating higher expression levels and blue indicating lower expression levels. The numbers under the color bars indicate specific changes in expression levels. (**B**) YLGB-1496 feeding group significantly increased gene expression in OP50, BL-99, YLGB-1496 feeding groups. Each group has three replicates, *n* > 1000, * *p* < 0.05, ** *p* < 0.01.

The structural molecule activity pathway, which is enriched with genes contributing to the structural integrity of complexes, was also analyzed. We compared the primary DEGs in this pathway between the YLGB-1496-treated group and the control group. As illustrated in Figure 5A, relative to the OP50 group, 14 genes were significantly upregulated in the YLGB-1496 group (*p* < 0.05), including 6 genes associated with cuticle development involved in collagen (*col-17*, *col-39*, *col-41*, *col-73*, *col-92*, and *col-107*), 7 genes associated with cuticle collagen coding (*dyp-4*, *dyp-7*, *dyp-13*, *sqt-1*, *sqt-2*, *rol-6*, and *ram2*), 1 genes associated with encoding ribosomal proteins (*rpl41.2*). Notably, the DEGs predominantly indicated that YLGB-1496 intervention enhances cuticle collagen synthesis in nematodes.

## 4. Discussion

This study utilized *C. elegans* as a model organism in conjunction with transcriptomic analysis to compare the anti-aging effects, anti-infective capabilities, and underlying mechanisms of the probiotic strains BL-99 and YLGB-1496. Experimental results demonstrated that feeding *C. elegans* with BL-99 and YLGB-1496 significantly extended the mean lifespan of wild-type nematodes by 10.96% and 12.14%, respectively, while also ameliorating age-related phenotypes and delaying the decline in pharyngeal pumping rate and locomotor capacity during aging. Furthermore, both strains enhanced nematode tolerance to *Staphylococcus aureus* infection, increasing lifespan by 5.90% and 14.38%, respectively, and significantly attenuating the age-associated deterioration in pharyngeal pumping and movement. In these assays, BL-99 and YLGB-1496 exhibited comparable anti-aging and anti-infective efficacies. However, transcriptomic analyses revealed that the molecular mechanisms underlying their effects were nearly distinct, with only 7.87% of differentially expressed genes overlapping between the two strains compared to the control group, and no shared pathways identified in GO enrichment analysis. Specifically, the BL-99 intervention uniquely augmented the nematode’s defense response against Gram-positive bacteria, whereas the YLGB-1496 intervention primarily enhanced the synthesis capacity of structural molecules, particularly collagen, in nematodes.

Numerous targets and pathways influence the lifespan of nematodes. Recent experimental evidence indicated that *Bifidobacteria* can extend nematode lifespan through various signaling pathways. For instance, *B. longum* strain BB68 enhanced nematode longevity by activating the *TIR-1-JNK-1-DAF-16* signaling pathway [25]. *B. infantis* modulated nematode lifespan via the *TOL-1*/TLR pathway [27]. Additionally, *B. animalis* subsp. *lactis* BPL1™ prolonged longevity in *C. elegans* through its lipoteichoic acid by an insulin/*IGF-1*-dependent mechanism [28]. Furthermore, cell wall components of *B. infantis* BI increased the average lifespan of *C. elegans* by activating *skn-1*, regulated by the p38 MAPK pathway. Given the complexity of the targets and pathways involved, we employed transcriptomic analysis to compare the action pathways of two strains.

The efficacy of bifidobacteria varies across species. These effects have been validated in various models, including mice, *Drosophila melanogaster*, and *C. elegans*. For instance, *B. adolescentis* has been shown to regulate catalase activity and host metabolism, improve osteoporosis and neurodegeneration in a mouse model of premature aging, and increase both health span and lifespan in *C. elegans* [29]. *B. longum* subsp. *longum* YS108R has been demonstrated to alleviate DSS-induced colitis through its anti-inflammatory properties, by protecting mucosal barrier integrity and maintaining gut microbiota homeostasis [7]. *B. animalis* subsp. *lactis* LKM512 mitigated obesity-associated inflammation and insulin resistance by modifying gut microbiota in high-fat diet-induced obese mice and prolongs the lifespan of the host [30]. In previous studies, it has been found that BL-99 and YLGB-1496 belong to different species of *Bifidobacterium*, which are clearly different in taxonomy, and the reported efficacy is not the same [22,23]. Similarly, in our study, although BL-99 and YLGB-1496 exhibited comparable lifespan-extending effects, transcriptomic analyses indicated that their mechanisms were fundamentally different.

In this study, the BL-99 groups exhibited significant enrichment in defense responses against Gram-positive bacteria, including the upregulation of lysozyme, saposin-like antimicrobial peptides, and c-type lectin family genes. The defense response against Gram-positive bacteria is critical for responding to external stimuli and maintaining health. This response is particularly significant for the elderly [31]. As Gram-positive bacteria, such as *S. aureus*, *S. pneumoniae*, *Listeria monocytogenes*, etc. *S. aureus* can lead to pneumonia, thereby increasing the risk of respiratory failure and heart failure [32]. *Listeria monocytogenes* poses a significant threat to the elderly and immunocompromised individuals, as it can infiltrate the body via the digestive and respiratory tracts, as well as through skin lesions [33]. Consequently, bolstering resistance to bacterial and viral infections is crucial for the elderly demographic. Enhancing anti-infective measures can substantially improve recovery rates and reduce mortality in this population. Research by Jane M. Natividad et al. demonstrated that *B. brevis* NCC2950 can stimulate the expression of antimicrobial peptides, thereby augmenting the defense mechanisms in mice against Gram-positive bacteria [34]. Furthermore, Miroslav Dinić et al. found that *Lactobacillus curvatus* BGMK2-41 activated the *PMK-1*/p38 MAPK immunity pathway, which extended the survival of *C. elegans* when exposed to *S. aureus* in nematode [35].

In our study, the intervention with BL-99 significantly upregulated the expression of *lys-4*, *lys-5*, *lys-6*, and *ilys-5*, thereby contributing to pathogen resistance. Lysozyme plays a crucial role in various physiological processes, including the elimination of pathogenic organisms, the processing of endocytosed nutrients, and antigen presentation [36]. The genes encoding lysozyme, specifically *lys-6*, *lys-4*, *lys-5*, and *ilys-5*, have demonstrated responsiveness to both pathogenic and non-pathogenic microorganisms, with a sequential decrease in sensitivity. Lysozyme exerts its antibacterial activity by degrading bacterial peptidoglycan through its enzymatic action [37]. Lysozyme can degrade bacterial peptidoglycan, enhancing antibacterial activity.

In our study, the BL-99 intervention significantly increased the expression of *spp-2* and *spp-3*, thereby playing a crucial role in combating pathogenic organisms. Saposin-like antimicrobial peptides constitute a group of polypeptides that play a significant role as antimicrobial agents in the innate immunity of *C. elegans* [38]. Among the genes encoding these peptides, *spp-2* and *spp-3* have been shown to enhance the organism’s resistance to infections [39]. These peptides can form pores in phospholipid vesicles, potentially facilitating interactions between the worms and microorganisms [40].

In our investigation, BL-99 intervention significantly elevated the expression levels of *clec-51*, *clec-163*, and *clec-169*, which are members of the C-type lectin gene family. According to existing literature, it is hypothesized that these genes play a pivotal role in enhancing immune function. The C-type lectin family constitutes a critical element of innate immunity in eukaryotic organisms [41]. An upregulation in the expression of C-type lectin family genes indicates that *C. elegans* may sustain their anti-aging phenotype by augmenting immune surveillance and maintaining protein homeostasis [42]. The secretion of C-type lectins can bolster resistance against pathogenic bacterial infections [43].

In this study, the YLGB-1496 treatment groups demonstrated significant enrichment in the enhancement of the stratum corneum and collagen structure, including the development of the collagen stratum corneum, as well as the synthesis and assembly of collagen. Collagen is integral to human health, particularly in the context of aging. Research by Yao Zhu et al. has demonstrated that the structural components of the stratum corneum may significantly influence developmental and aging processes [44]. A reduction in collagen can compromise the skin barrier function in elderly individuals, leading to thinning of the epidermis and dermis and diminished antibacterial capacity of the skin [45]. The epidermis and eggshell of *C. elegans* are abundant in collagen, keratin, and chitin, which confer resistance against the virulence factors of fungal hydrolytic enzymes [46]. The ability to enhance collagen synthesis can contribute to the increased longevity of nematodes [20].

In our research, intervention with YLGB-1496 markedly upregulated the expression of the *col-92* gene, thereby enhancing the epidermal barrier. Collagen is crucial for maintaining the permeability barrier of the stratum corneum, thereby preventing the invasion of bacteria such as *S. aureus* through the epidermis. As the primary physical defense mechanism in nematodes, the stratum corneum is instrumental in preventing the invasion, accumulation, and dissemination of pathogens to internal tissues such as the intestine or epidermis, thus mitigating cell damage and mortality due to infection. Valentina Taverniti et al. found that multiple strains of probiotics can improve intestinal barrier function and reduce inflammation in mice [47]. The collagen-encoding gene *col-92* has been identified as playing a significant role in the defense against *Bacillus thuringiensis* [48].

The synthesis of collagen, which constitutes the epidermis, is a complex process that spans the entire life cycle of *C. elegans* [49]. The regulation of collagen synthesis by these genes may induce alterations in the exoskeleton, thereby influencing the growth and development of *C. elegans* [50]. The *dpy-13* gene, which encodes epidermal collagen, is extensively utilized as a marker for aging in the evaluation of tissue integrity and lifespan in nematodes. Research by Nidhi Thakkar et al. demonstrated that the root extract of *Withania somnifera* can significantly upregulate the expression of collagen-related genes, such as *dpy-13* in senescent nematodes [51], corroborating the upregulation findings of the *dpy-13* gene in this study.

The maintenance of the epidermal permeability barrier through collagen is crucial for the anti-infective defense of nematodes. Increased infiltration of morphologically abnormal cells renders nematodes more vulnerable to infection by *S. aureus*, conversely, enhancing the barrier can improve survival rates. Collagen encoded by the *rol* family genes is essential for maintaining the penetration barrier of the cuticle, thereby preventing bacterial invasion, such as by *S. aureus*, through the epidermis.

Collectively, our results demonstrate that the intervention with YLGB-1496 significantly upregulated the expression of the gene *rol-6*, playing a crucial role in maintaining the epidermal permeability barrier through collagen synthesis. Research has demonstrated that the downregulation of certain genes can result in morphological abnormalities and increased infiltration of the cuticle, rendering nematodes more susceptible to *S. aureus*. Conversely, upregulation of these genes enhances survival rates by fortifying the barrier [52].

## 5. Conclusions

This study compared the effects of two *Bifidobacterium* strains on pathogen resistance and lifespan extension in *C. elegans*. Although both *Bifidobacterium animalis* subsp. *lactis* BL-99 and *Bifidobacterium longum* subsp. *infantis* YLGB-1496 demonstrated similar capabilities in combating infections and extending lifespan, their mechanisms of action were notably distinct. The BL-99 strain enhanced nematode resistance to Gram-positive pathogens by upregulating the expression of lysozyme, saposin-like antimicrobial peptides, and c-type lectin family genes, thus enhancing the anti-infection ability and prolonging the life span. In contrast, the YLGB-1496 strain improved the epidermal permeability barrier by upregulating genes associated with collagen synthesis and assembly, thereby contributing to pathogen resistance and anti-aging effect. This study provides new evidence for the species-specific effects of bifidobacteria in pathogen resistance and lifespan extension.

## Data Availability

The original contributions presented in this study are included in the article/Supplementary Material. Further inquiries can be directed to the corresponding author.

## Supplementary Materials

The following supporting information can be downloaded at: https://www.mdpi.com/article/10.3390/microorganisms13122861/s1, Table S1: All strains used in this study.
