# Supplementary material for: Comparative Mechanisms of Different Bifidobacteria in Combating Pathogen Infection and Prolonging the Lifespan in Caenorhabditis elegans
Source: Microorganisms. 2025 Dec 16;13(12):2861. doi: 10.3390/microorganisms13122861 (PMC12735594; doi:10.3390/microorganisms13122861)
Supplement: Supplementary file 1 [file microorganisms-13-02861-s001.zip › microorganisms-3955938-supplementary.pdf]

Table S1. All strains used in this study.

Strains	Genotype	Source
N2	Wild-type	Professor Yanling Hao's laboratory, China Agricultural University
OP50	*Escherichia coli* OP50	Professor Yanling Hao's laboratory, China Agricultural University
Staphylococcus aureus	*Staphylococcus aureus* ATCC25923	Professor Yanling Hao's laboratory, China Agricultural University
BL-99	*Bifidobacterium animalis subsp. lactis* BL-99	the National Technology Innovation Center for Dairy in Hohhot, China.
YLGB-1496	*Bifidobacterium longum subsp. infantis* YLGB-1496	the National Technology Innovation Center for Dairy in Hohhot, China.
